# CDK 4/6 inhibitors for the treatment of meningioma

**DOI:** 10.3389/fonc.2022.931371

**Published:** 2022-07-22

**Authors:** Jacob S. Young, Reilly L. Kidwell, Allison Zheng, Alex F. Haddad, Manish K. Aghi, David R. Raleigh, Jessica D. Schulte, Nicholas A. Butowski

**Affiliations:** ^1^ Department of Neurological Surgery, University of California San Francisco, San Francisco, CA, United States; ^2^ Department of Radiation Oncology, University of California San Francisco, San Francisco, CA, United States; ^3^ Division of Neuro-Oncology, University of California San Diego, San Diego, CA, United States; ^4^ Department of Neuroscience, University of California San Diego, San Diego, CA, United States; ^5^ Division of Neuro-Oncology, University of California San Francisco, San Francisco, CA, United States

**Keywords:** CDK inhibitor, meningioma, cell cycle dysregulation, clinical trials, molecular profiling and subtyping

## Abstract

Meningiomas are the most common non-metastatic brain tumors, and although the majority are relatively slow-growing and histologically benign, a subset of meningiomas are aggressive and remain challenging to treat. Despite a standard of care that includes surgical resection and radiotherapy, and recent advances in meningioma molecular grouping, there are no systemic medical options for patients with meningiomas that are resistant to standard interventions. Misactivation of the cell cycle at the level of CDK4/6 is common in high-grade or molecularly aggressive meningiomas, and CDK4/6 has emerged as a potential target for systemic meningioma treatments. In this review, we describe the preclinical evidence for CDK4/6 inhibitors as a treatment for high-grade meningiomas and summarize evolving clinical experience with these agents. Further, we highlight upcoming clinical trials for patients meningiomas, and discuss future directions aimed at optimizing the efficacy of these therapies and selecting patients most likely to benefit from their use.

## Introduction

Meningiomas are the most common primary intracranial tumor, and although the vast majority of meningiomas are considered Grade 1 tumors by the World Health Organization (WHO) and can be managed effectively, between 20-30% of cases are considered Grade 2 or 3 and prove challenging to treat. Surgery and radiotherapy are the therapeutic foundation of meningioma management, with no chemotherapeutic agents currently approved for these tumors (1). While there has been significant recent advances in the meningioma prognostication and classification using genomic and DNA methylation classifications, less progress has been made in their therapeutic treatment (2–9). Unfortunately, when these high-grade lesions recur and/or are found in regions along the skull base that make complete resection challenging, they often cause significant morbidity and ultimately prove to be fatal for patients. In this review, we describe the therapeutic rationale and preclinical/clinical evidence for small molecule inhibitors that target key cell cycle regulators, specifically cyclin dependent kinase (CDK) proteins, in the treatment of meningioma.

## CDK 4/6 role in tumorigenesis

**Figure 1:**
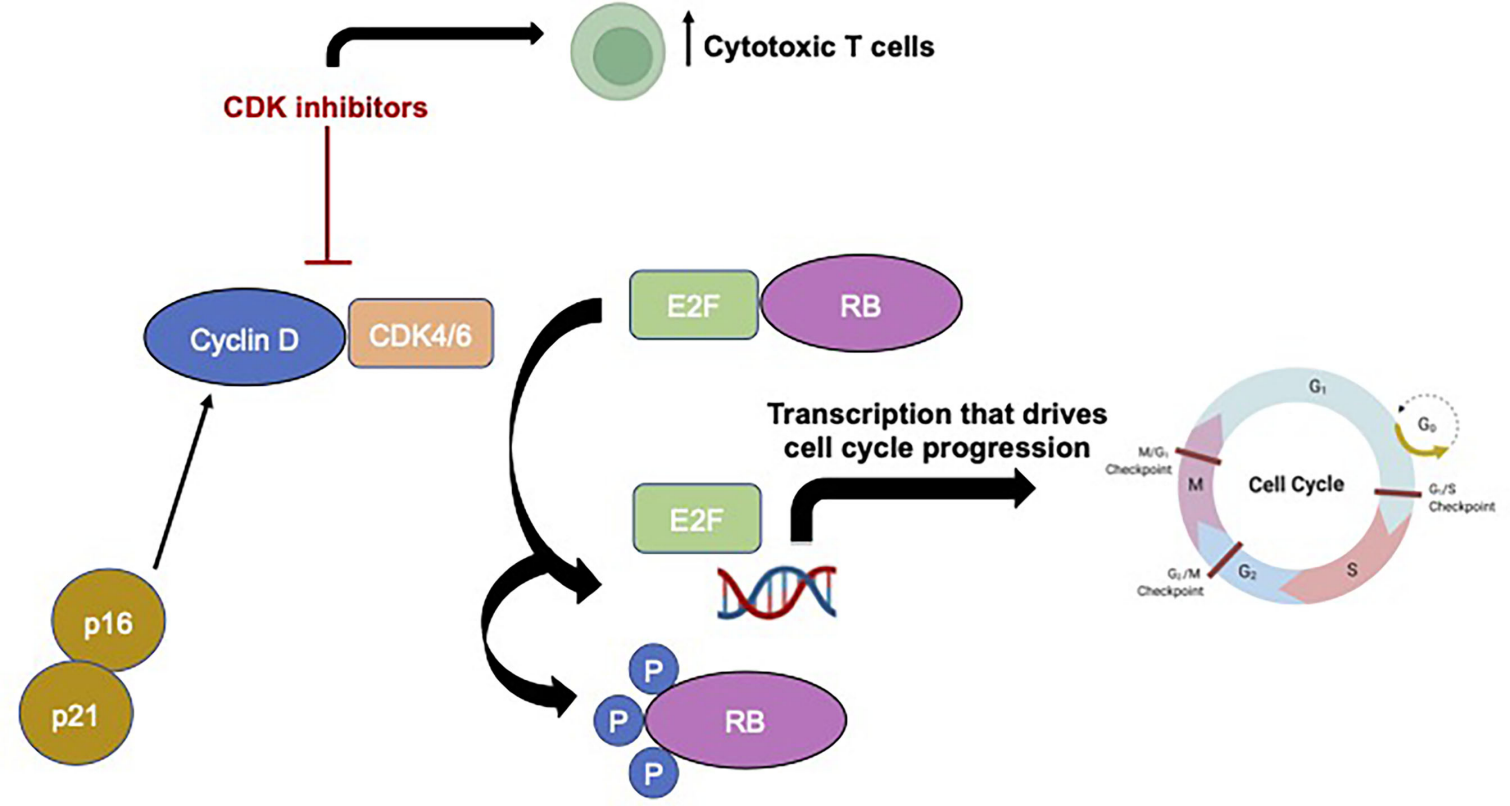
Schematic showing basic cyclin-CDK signaling pathway and mechanism of action of CDK inhibitors Made in BioRender.

In non-pathological states, the process of cell division requires cells to progress through a series of highly regulated stages in sequential order, termed the cell cycle, and numerous checkpoints are present to prevent a cell from dividing in the absence of growth factors or in the presence of DNA damage (10). However, dysregulation of these cell division processes and uncontrolled cellular proliferation is a hallmark of cancer (10). CDKs interact with cyclin proteins to regulate this transition from one stage to the next, and, unsurprisingly, increased levels of these CDKs and their regulators, like FOXM1, are commonly observed in cancers such as meningiomas (11–14). CDK4 and 6 are two structurally similar cell cycle regulators that ultimately stimulate a cell forward in cell division to the S phase from G0/G1 (see **Figure 1** for schematic of cyclin-CDK pathway). The downstream targets of CDK4/6 include the classic, canonical tumor suppressor protein, retinoblastoma (Rb), and following phosphorylation of Rb by CDK4/6, the transcription factor E2F is able to initiate DNA synthesis and the S phase of cell division (15). Inhibitors of CDK, termed cyclin-dependent kinase inhibitors (CKIs), regulate CDK activity and decreased expression of these regulatory proteins is frequently observed in many cancers, with p16, which is encoded by the gene *CDKN2A*, being the most well characterized CKI. Furthermore, dysregulation of p16, CDK6, and pRB protein have all been associated with recurrence in atypical meningiomas (16) and homozygous deletions of the *CDKN2A/B* gene has also been associated with early meningioma recurrence (17). Given their position as relatively upstream regulators of these crucial cell cycle pathways, CDK4/6 specific inhibitors have become very attractive cancer therapeutic agents.

## Development of CDK inhibitors for treatment of malignancies

Pan-CDK inhibitors were first developed over three decades ago, but their therapeutic potential was thwarted by severe toxicities, and now more specific inhibitors have mostly replaced these early pan-CDK inhibitors (18). There are currently three FDA-approved CDK4/6 specific inhibitors available in the United States: Palbociclib, Ribociclib, and Abemaciclib, each with their own specific pharmacokinetics and toxicities. These agents have been used as monotherapy or in combinatorial approaches with other therapies for the treatment of various cancer types.

Breast cancer was one of the first malignancies where CDK inhibitors were utilized given promising preclinical data demonstrating reliance on CDK signaling during breast cancer tumorigenesis. All three specific inhibitors listed above demonstrated efficacy when used as treatment of estrogen receptor (ER)-positive breast cancer in combination with anti-estrogen therapy, replacing the previous gold standard of anti-estrogen therapy alone for ER-positive breast cancers (19–21). Palbociclib has been shown to be efficacious in other hormone receptor (HR)-positive breast cancer cell lines and is the only agent that can be used for perimenopausal and premenopausal women (22). When combined with an ER antagonist, Palbociclib significantly improved progression free survival, but not overall survival, in HR+ breast cancer (23, 24). Abemaciclib was also found to be safe and have some benefit as a single agent in HR+ breast cancer patients (25). Finally, Ribociclib may have a synergistic effect when used with an ER antagonist, and was found to improve PFS and overall response rate in patients with HR+/HER2- advanced breast cancer (26).

These examples of varying therapeutic efficacy to the different CDK4/6 inhibitors in breast cancer demonstrates the importance of finding biomarkers for tumor sensitivity to these agents. While hormone receptors may prove to be a powerful biomarker for breast cancer responsiveness, other markers are needed for other tumor types. CCND1 amplification and loss of p16 expression may indicate sensitivity to CDK inhibitors in breast cancer, although results are conflicting in the literature (19, 27). Another group of proteins, termed D-cyclin activating features (DCAFs), have also been associated with CDK4/6 inhibitor sensitivity (28). Furthermore, it is equally important to understand how resistance develops to CDK4/6 inhibitors, which seems to be common after prolonged treatment with these agents (29). As CDK4/6 inhibitors are trialed for patients with aggressive meningiomas, it will be important to design clinical trials incorporating window-of-opportunity strategies to obtain tissue for pharmacokinetics, pharmacodynamics, and biomarker analysis from treated patients.

There may be synergistic lethality in targeting CDK4/6 targets in combination with other signaling pathways, particularly those that interact with cell cycle regulation pathways. Other signaling pathways interact with CDK4/6 targets, such as the PI3K-AKT-mTOR and the RAS-RAF-MEK-ERK pathways, may also provide potential therapeutic targets that synergize with CDK4/6 inhibitors. For example, inhibitors of PI3K pathway proteins have been effective in preclinical breast cancer, mesothelioma, and head and neck cancer models when combined with CDK4/6 inhibition (30–32). Mutations in KRAS, NRAS, BRAF genes also lead to activation of the RAS-RAF-MEK-ERK pathway, and treatment with CDK4/6 inhibitors may have a synergistic effect when used with inhibitors of the RAS pathway (33). Like the PI3K pathway, RAS pathway inhibition alters mTOR levels to impact cell proliferation (34–36). Further investigation is needed to determine if inactivation of these overlapping signaling pathways will help prevent resistance to these agents and if there is a role for combinatorial strategies for the treatment of meningioma patients.

## Preclinical evidence for CDK inhibitors in meningiomas

As mentioned above, cyclin overexpression has been associated with increased grade and risk of recurrence in meningioma (6, 37–40). Prior to the advent of CDK inhibitors, early preclinical studies utilized targeted small interfering RNA (siRNA) to inhibit CDK. Cheng et al. were one of the first groups to show that targeting cyclin D1 levels decreased cell proliferation, cell viability, and halted tumor cell invasion in malignant meningioma (41). Cyclin D1 knockdown was also shown to decrease antiapoptotic proteins such as survivin and Bcl-2, increasing time in G0/G1 phase and causing cell cycle arrest. siRNA targeting of cyclin D1 also diminished meningioma cell invasion *via* suppression of extracellular matrix metalloproteinases *in vitro*. This work opened the door for investigation of pharmacologic CDK inhibitors as therapeutic agents for meningioma.

Subsequent pre-clinical studies revealed anti-tumor effects for CDK inhibitors in various *in vitro* and *in vivo* meningioma models. The majority of studies utilized Palbociclib, which is the most frequently used CDK4/6 inhibitor in cancer clinical trials (42). Das et al. found Palbociclib induces G1 cell-cycle arrest and tumor cell apoptosis in a radiation-induced malignant meningioma model (43). Using Grade 1 and Grade 3 meningioma cell lines, Palbociclib treatment inhibited the expression of CDK4/6 and downstream E2F transcription factor, resulting in dramatic reduction of pRB and reduced cell proliferation. Treatment with 14 days of Palbociclib (10mg/kg) plus radiation (6 Gy) reduced total tumor volume in an *in vivo* subcutaneous mouse meningioma xenograft model. Work by Horbinski et al. further supported Palbociclib-induced suppression of pRb and cell proliferation *in vitro*, specifically in p16-/Rb+ meningioma cell lines (44). In contrast, p16+/Rb- cell lines were resistant to both radiation and CDK inhibition. This study also demonstrated combination therapy with radiation and Palbociclib significantly delayed tumor growth and prolonged overall survival in mouse xenograft models compared to ether treatment alone. Interestingly, this effect was primarily attributed to decreased cell proliferation, as histological analyses failed to demonstrate any difference in apoptosis or cell death.

Given CDK4/6 inhibitors are thought to be largely cytostatic (45), rather than cytotoxic when used as monotherapy, and there are still toxicities associated with these agents (46, 47), there is significant preclinical interest in combinatorial strategies and/or novel agents that may be cytotoxic. One example, TG02 (SB1317) is an orally available, multi-cyclin-dependent kinase inhibitor of CDK 1,2,5,7 and 9. As specific inhibition of CDK9 has been shown to induce downstream depletion of key oncoproteins including MCL-1 and c-MYC, targeting this CDK protein has also become of interest as a cancer therapy (48, 49). Von Achenbach et al. examined the effects of TG02 in primary patient-derived meningioma cell lines classified as benign, intermediate, or malignant by DNA methylation profiling and found dose-dependent inhibition of cell proliferation across cultures, without significant induction of apoptosis (50). Importantly, cell lines classified as malignant were overall more sensitive than those considered benign.

As mentioned above, there has significant interest in molecular profiling to improve patient selection and clinical response rates to CDK inhibition in patients with recurrent meningioma. Using DNA methylation profiling of 565 primary meningioma samples, Choudhury et al. identified three DNA methylation groups with distinct clinical outcomes and biological drivers: (A) Merlin-intact, (B) Immune-enriched, and (C) hypermitotic, and the latter group was notably had a loss of the endogenous CDK4/6 negative regulator, *CDKN2A/B* (51). Exposing patient cells from this group to the known CDK4/6 inhibitors Abemaciclib, Palbociclib, and Ribociclib resulted in growth attenuation across cell culture, organoid, and xenograft models. Specifically, *in vivo*, CDK4/6 blockade diminished pRb expression, inhibited cell proliferation, and prolonged overall survival. This study highlights the role DNA methylation profiling may play as a clinical tool to stratify meningioma patients for molecular treatments.

Agents that indirectly alter the CDK pathway are also being explored as potential meningioma therapies. For example, Negroni et al. found upregulation of the zinc finger transcription factor GATA binding protein 4 (GATA-4) in high grade meningioma primary patient samples, which resulted in overexpression of cyclin D (52). Accordingly, administration of NSC140905, a small molecule inhibitor of GATA-4 reduced expression of cyclin D1 and diminished meningioma cell viability *in vitro*. Another group is targeting the eukaryotic initiation factor 4F complex (eIF4F), which regulates the translation of many pro-oncogenic proteins like MYC and cyclins in various cancers (53). Oblinger et al. found elevated levels of eIF4A in primary meningioma samples and showed this protein to be a driver of tumor cell proliferation *via* induction of downstream cyclin-mediated signaling (54). Treatment of cells with silvestrol, an inhibitor of eIF4A, resulted in reduction of cyclins D1 and E1, and G2/M phase arrest. Although these inhibitors are further from clinical trials than the more established CDK inhibitors, these agents pose a novel and promising therapeutic possibility for targeting cyclin-mediated signaling in meningioma.

## Meningioma tumor microenvironment on CDK inhibitors

The importance of the brain tumor microenvironment has blossomed in the era of immunotherapy, particularly for highly immunosuppressive tumors like glioblastoma. Given meningiomas ability to invade both brain and bone, early research investigating the meningioma microenvironment focused on specific extracellular matrix components, like matrix metalloproteinase expression (55). However, more recent research has begun to elucidate the importance of immune cells in the microenvironment. For example, new classification schema have emerged based on tumor DNA methylation signatures, with one category of meningiomas considered “immune-enriched” (56). Moreover, in addition to having more immunosuppressive infiltrating immune cells, higher-grade meningiomas appear to express more PD-L1 on tumor cells and tumor-infiltrating CD68+ macrophages (57, 58). Indeed, a large percentage of the meningioma microenvironment consists of CD45+ immune cells (59), with the macrophage population making up the largest percentage of this compartment (60).

Interestingly, the mechanism of action of CDK inhibitors is likely not as simple as once thought. In addition to the direct effect on cycling tumor cells, CDK4 influences the composition of cells in tumor microenvironment and inhibition of this pathway results in changes in the tumor-infiltrating immune cell populations (61). In breast cancer models, CDK inhibition increased antigen presentation and increased the number of cytotoxic T cells in the tumor microenvironment while simultaneously reducing the number of immunosuppressive regulatory T cells (62). Currently, there is very little literature regarding the impact of CDK inhibition on the meningioma tumor microenvironment and even less is known how the meningioma microenvironment contributes to treatment resistance or efficacy.

## Clinical trials using CDK inhibitors

To date, one clinical trial investigating CDK inhibitors for meningioma has been one completed and four additional trials are ongoing, for which results have yet to be published (**Table 1**). Many of these trials include multiple central nervous system (CNS) tumors, and the number of meningioma patients enrolled is currently unknown.

**Table 1:** Ongoing CDK inhibitor trials for meningioma.

Study title	Drug	Phase	Patient population	Sponsor	Status	Trial registration no.
PBTC-042: Palbociclib Isethionate in Treating Younger Patients with Recurrent, Progressive, or Refractory Central Nervous System Tumors	Palbociclib Isethionate	I	Recurrent Rb1+ childhood grade III meningioma; other Rb1+ CNS tumors	Pediatric Brain Tumor Consortium (Collaborator: NCI)	*Terminated* (Primary objective complete; MTD determined)	NCT02255461
SJDAWN: Phase 1 Study Evaluating Molecularly-Driven Doublet Therapies for Children and Young Adults with Recurrent Brain Tumors	Stratum B: Ribociclib + Trametinib	I	Recurrent anaplastic meningioma; other CNS tumors	St. Jude Children’s Research Hospital (Collaborator: Novartis)	*Recruiting*	NCT03434262
Ribociclib (LEE011) in Preoperative Glioma and Meningioma Patients	Ribociclib	0/II	Preoperative; Rb+ or non-Rb-mutated recurrent grade II/III meningioma; glioma	Nader Sanai (Collaborators: Novartis, Ivy Brain Tumor Center, Barrow Neurologic Institute)	*Recruiting*	NCT02933736
A071401: Vismodegib, FAK Inhibitor GSK2256098, Capivasertib, and Abemaciclib in Treating Patients with Progressive Meningiomas	Cohort D: Abemaciclib	II	Meningioma with CDK4, CDK6, CDKN2A, CCND1, CCND2, CCND3, or CCNE1 alterations	Alliance for Clinical Trials in Oncology (Collaborators: NCI, GlaxoSmithKline, Genentech, Brain Science Foundation)	*Recruiting*	NCT02523014
MSK 17-261: Abemaciclib (LY2835219) in Patients with Recurrent Primary Brain Tumors	Cohort C: Abemaciclib	II	Recurrent meningioma	Memorial Sloan Kettering Cancer Center (Collaborator: Eli Lilly and Company)	*Recruiting*	NCT03220646

PBTC-042 was a phase I open-label dose-escalation trial to assess the maximum tolerated dose (MTD) and pharmacokinetics of daily oral PD-0332991 (Palbociclib isethionate) in Rb1+ recurrent, progressive, or refractory primary CNS tumors in young adults (NCT02255461). Secondary endpoints included evaluation of efficacy, genetic profiling of tumor samples, and further exploration of pharmacokinetic (PK) parameters. The study was terminated upon completion of primary endpoints and identification of the MTD, although detailed results have not yet been presented or published and it is unclear how many, if any, were meningioma patients. Outcomes data on ClinicalTrial.gov indicate a MTD of 75mg/m2 was identified, with hematologic toxicities, including anemia, neutropenia, and leukopenia predominantly being dose-limiting. Other common toxicities reported in this study included nausea, constipation, diarrhea, fatigue, and transaminitis, although these were not considered serious adverse events. There was also one serious non-hematologic adverse event of dehydration, and these non-hematologic toxicities are one reason these agents have been poorly tolerated by patients and are not more widely used clinically to date. No patients showed objective responses (defined as complete or partial response).

Currently recruiting trials have focused on the CDK4/6 inhibitors Ribociclib (LEE011) and Abemaciclib (LY2835219), the latter of which is distinguished by a shorter half-life and a slightly higher affinity for CDK4 (46). SJDAWN is a Phase 1 dose-escalation clinical trial exploring molecularly driven doublet (or combinatorial) therapies unique to a patient’s specific tumor type (NCT03434262). Patients who tolerate the drug combination are eligible for an expansion cohort to assess for early efficacy. Stratum B of this trial includes patients with recurrent or refractory anaplastic meningioma treated with combination Ribociclib and the MEK inhibitor Trametinib. Primary endpoints include determination of MTD and PK analysis and secondary outcomes include response rate and duration of objective response. The trial is currently ongoing, and no interim results have been reported to date.

Another ongoing study is investigating single-agent Ribociclib in the adult population as a phase 0/2 non-randomized open-label trial evaluating preoperative dosing of oral Ribociclib in patients with Rb+ or non-Rb-mutated recurrent WHO Grade 2/3 meningioma or high-grade glioma (NCT02933736). In this trial, patients receive 900mg of Ribociclib daily for 5 days prior to surgical resection and endpoints include evaluation of PK, PD, and tissue analyses for signs of any preliminary clinical response. PD analysis includes assessment of Rb and FOXM1 phosphorylation as markers of halted cellular progression from G1 to S phase (63). Interim results reported a median CSF concentration of ribociclib was 0.25 μM and tumor tissue concentration of unbound ribociclib 1.36 μM, and 4 out of 8 patients had a positive PK and PD tumor response (defined as unbound ribociclib concentration > 5-fold *in vitro* IC50 (0.04 μM) and >20% decrease in pRB levels, respectively) (64). These patients defined as PK/PD responders were subsequently enrolled in an exploratory Phase 2 cohort of continuous Ribociclib therapy (600mg daily for 3 weeks/1 week off). At 1 year on therapy, 2 of 4 patients were assessed to have a partial response (PR) by RANO criteria. Overall progression-free survival (PFS) was >12 months in 3 of 4 patients, and >23 months in the 4^th^ patient. Given continuous Ribociclib in other solid tumors has been shown to have an acceptable safety profile, there is excitement for the final results of this ongoing study (25). Although the reThis study also showcases the importance of performing more Phase 0 and “window-of-opportunity” studies to confirm PK/PD for trials investigating CDK inhibitors for meningioma (65).

The remaining two ongoing studies aim to examine the efficacy of twice daily dosing of oral Abemaciclib. The only trial to enroll meningioma patients alone is A071401, a Phase 2 trial of SMO/AKT/NF2/CDK inhibitors in patients with progressive meningiomas harboring corresponding mutations in the respective signaling pathway (NCT02523014). Patients are considered eligible for Abemaciclib if molecular testing is positive for alterations in CDK4, CDK6, CDKN2A, CCND1, CCND2, CCND3, or CCNE1, with primary endpoints including PFS and response rate by Macdonald criteria. To date, interim results have only been reported for the FAK inhibitor cohorts but have not been described for the ongoing Abemaciclib group (66). The second investigational study testing this agent is MSK 17-261, a Phase 2 open-label, non-randomized study of Abemaciclib in patients with recurrent primary brain tumors (NCT03220646), including patients with recurrent meningiomas. Dosing is 200mg of Abemaciclib twice a day, which follows the MTD established in the Phase 1 trial which included patients with glioblastoma, breast cancer, non-small cell lung cancer, and other solid tumors (67). Recent interim results suggest promising early efficacy data for the subset of recurrent meningioma patients, although full results have yet to be published (68).

## Future directions

As mentioned, one concern with the use of CDK4/6 inhibitors is the development of resistance mechanisms to these therapies through quasi-redundant or alternative signaling pathways, which has been reported in breast cancer and medulloblastoma patients receiving CDK inhibitor monotherapy (12). Daggubati et al. found that in Hedgehog-associated medulloblastoma, decreased ribosomal protein expression in response to CDK inhibitor treatment caused ER stress and activated the unfolded protein response, which ultimately upregulated production of sterol lipids that activate the Smoothened (SMO) to sustain the Hedgehog signaling pathway despite cell cycle attenuation (69). Interestingly, the authors found that combinatorial therapies with CDK inhibitor and a small molecule that inhibited the production of these SMO-activating lipids was able to effectively block cancer cell growth and may help overcome resistance to monotherapy. Additional studies identifying resistance mechanisms to these inhibitors will be critical to translating preclinical successes to durable responses for patients in the clinic. Finally, given the difficulty patients have tolerating these agents, local delivery strategies such as convection enhanced delivery or approaches to improve drug concentration in the tumor such as blood brain barrier disruption *via* focused ultrasound should be explored for these therapies.

## Conclusions

Patients with high-grade meningiomas face a difficult prognosis with no good systemic treatments available. Cell cycle regulators are commonly dysregulated in many cancers, including meningiomas, and represent a potential treatment strategy. Preclinical evidence supports the use of CDK4/6 specific inhibitors, Palbociclib, Abemaciclib, and Ribociclib, as potential therapeutic agents for meningioma patients and these agents are actively being explored in ongoing clinical trials. Future work identifying response biomarkers and mechanisms of resistance are needed to better select patients for these agents and improve their efficacy and durability.

## Author contributions

JY, RK, and AZ drafted the manuscript. JY, JS, and NB organized and designed topic for review, all authors critically reviewed manuscript. All authors contributed to the article and approved the submitted version.

## Funding

JY is supported by the Neurosurgery Research and Education Foundation Research Fellowship, the Glioblastoma Foundation Neil Peart Research Award, the Chan-Zuckerberg Biohub Physician Scientist Fellowship, and the ASCO Conquer Cancer Young Investigator Award in Drug Development. DR is supported by the UCSF Physician Scientist Scholar Program, the UCSF Wolfe Meningioma Program Project, and NIH grant R01 CA262311.

## Conflict of interest

The authors declare that the research was conducted in the absence of any commercial or financial relationships that could be construed as a potential conflict of interest.

## Publisher’s note

All claims expressed in this article are solely those of the authors and do not necessarily represent those of their affiliated organizations, or those of the publisher, the editors and the reviewers. Any product that may be evaluated in this article, or claim that may be made by its manufacturer, is not guaranteed or endorsed by the publisher.
